# Reversible Pulmonary Hypertension due to Combined Fistula between the Left Anterior Descending Artery (LAD) and Pulmonary Artery and Severe Stenosis of the LAD

**DOI:** 10.1155/2021/6629684

**Published:** 2021-03-04

**Authors:** N. Schizas, V. Patris, S. Giannaraki, G. Ifanti, I. Anagnostopoulos, M. Argiriou

**Affiliations:** ^1^Cardiovascular and Thoracic Department of Evangelismos General Hospital, Athens, Greece; ^2^Anesthesiology Department of Evangelismos General Hospital, Athens, Greece; ^3^Radiology Department of Evangelismos General Hospital, Athens, Greece; ^4^Cardiology Department of G. Gennimatas General Hospital, Athens, Greece

## Abstract

Coronary artery fistulas are usually diagnosed accidentally without the presence of any symptoms. On the other hand, the combination of fistula between the left anterior descending artery (LAD) and pulmonary artery (PA) and severe stenosis of the LAD, as in this case report, is a potential life-threatening condition. A 72-year-old patient was treated surgically after being diagnosed with fistula between the LAD and PA, severe stenosis of the LAD, and severe pulmonary hypertension. In following paragraphs, the case of this man and significant issues regarding the development and management of coronary artery fistulas are analyzed.

## 1. Introduction

Fistula between coronary arteries and pulmonary artery is a rare entity which in most cases is diagnosed in acute phase in patients presented with chest pain (40.39%) and dyspnea (26.25%) [1]. In this case report, a 72-year-old man was diagnosed with combined fistula between the left anterior descending artery (LAD) and pulmonary artery (PA) and severe stenosis of the LAD while pulmonary hypertension was present.

## 2. Case Report

**Figure 1:**
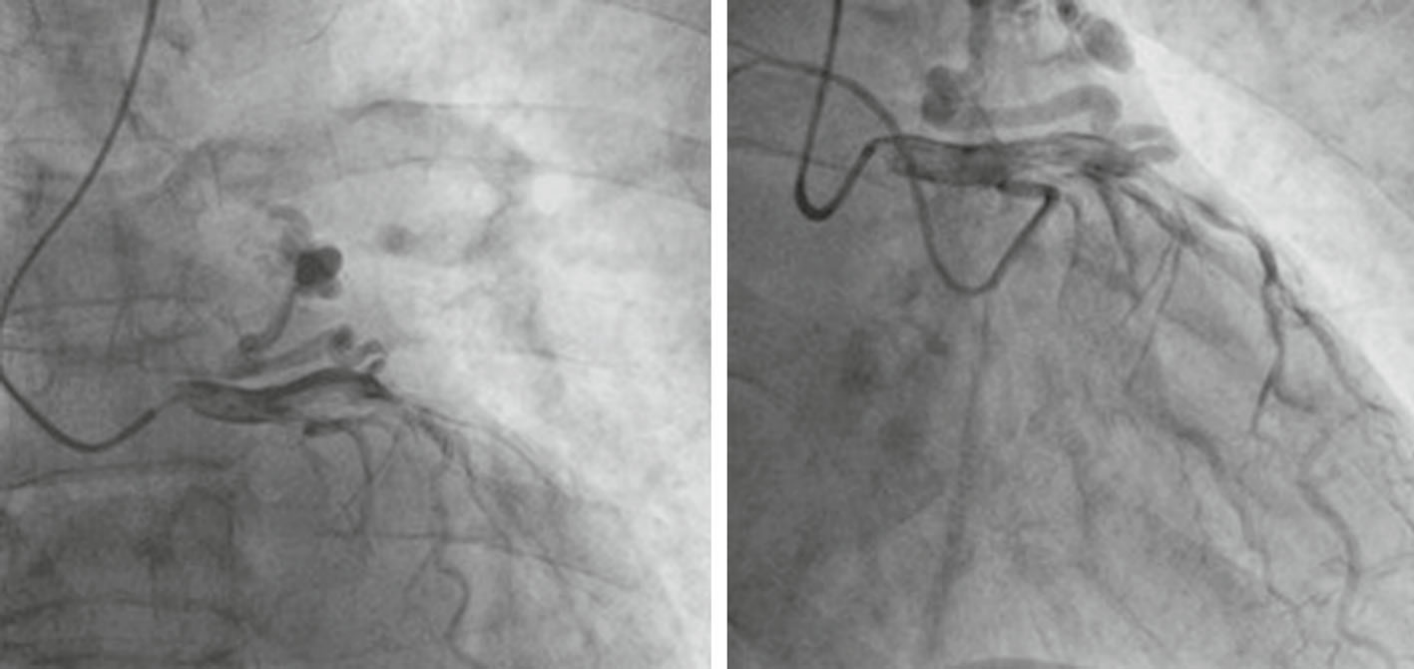
Pictures from the coronary angiography depicting the presence of fistula between the LAD and pulmonary artery.

A 72-year-old man was referred to our hospital due to unstable angina and dyspnea. The symptoms initiated a few hours before, and his medical background consisted of hypertension. The clinical examination revealed significant edema of both lower extremities, and there were no specific abnormalities at the electrocardiogram. The heart echo showed normal systole of the left ventricle with ejection fraction 65%, no pathological findings as regards the function of the heart valves, and increased pulmonary artery systolic pressure (PASP) which was estimated 55 mmHg while the volume of the right ventricle was 105 ml. As the angina persisted, the patient was submitted to a coronary angiography. The examination revealed a fistula between the left anterior descending artery (LAD) and pulmonary artery (PA) and in proximity to this a distal severe stenosis of the LAD (approximately 80%) (Figure 1). Moreover, a significant stenosis (70%) at the ostium of obtuse marginal artery (OM) was detected.

**Figure 2:**
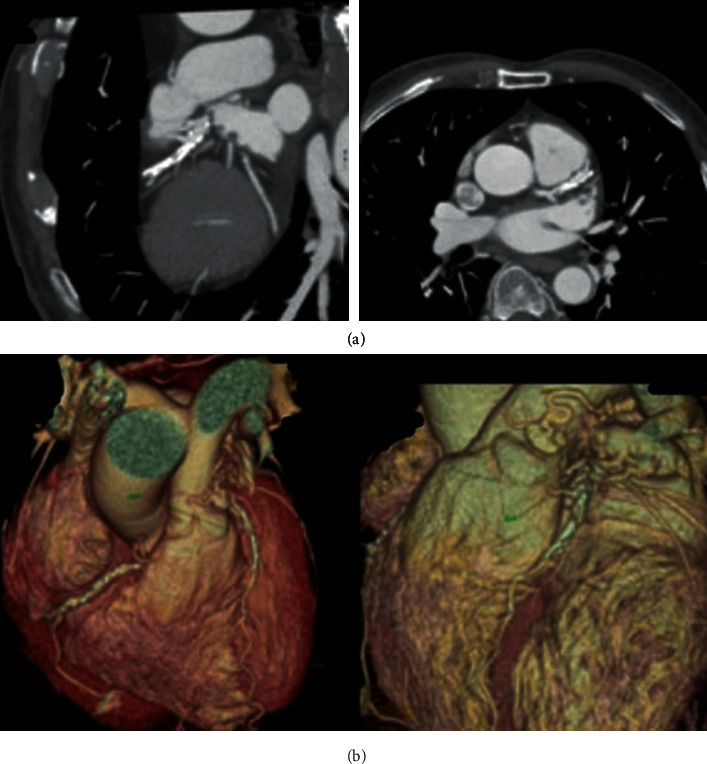
Depictions from coronary CT showing the LAD-PA fistula ((a) typical vertical sections and (b) 3D reconstruction images).

A coronary CT angiography was performed in order to verify this finding and to obtain more specific information (Figure 2). At this point, it should be mentioned that the options examined were two, the surgical approach and the percutaneous transcatheter intervention. The percutaneous intervention could be performed with the deployment of a large stent on the LAD in order to treat the stenotic lesion and at the same time cover the fistula's ostium, as reported in the literature [2], or the placement of vascular plug. After assessment of this information and shared decision-making of the heart team of our hospital, a surgical intervention was decided in the thought that multiple vessels had to be revascularized, acute events during the percutaneous intervention were possible, and a permanent solution would be the best option as the patient's physical status permitted an open surgery.

**Figure 3:**
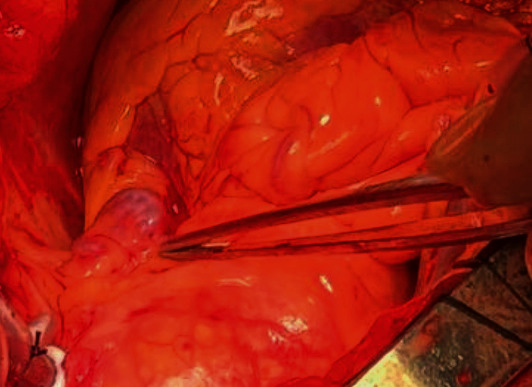
Intraoperative picture depicting the fistula between the LAD and PA (at the top of the surgical forceps).

The patient was submitted to a typical median sternotomy with aortic and atrial cannulation. After harvesting the grafts (left internal mammary (LIMA) and saphenous vein (SVG)), we proceeded with pump procedure. The fistula between the LAD and PA approximately 5 cm was identified, and we meticulously investigated the operative field in order to exclude the case of existence of more fistulas (Figure 3). This fistula was the only one and was ligated from both sides. After the completion of the ligation of the fistula, the coronary artery bypass grafting (CABG) was performed (LIMA to LAD and SVG to OM1).

The patient was hospitalized for 6 days with an uneventful postoperative rehabilitation and was discharged on the 7^th^ day. Before discharge, a heart echo showed satisfying function of the left ventricle (ejection fraction 65%), PASP was estimated 35 mmHg, and the volume of the right ventricle was 85 ml. In the follow-up examination after 3 months, the PASP estimation was 30 mmHg while the other parameters remained unchanged.

## 3. Discussion

Fistula between the LAD and PA is the most common communication between coronary arteries to PA with its incidence ranging from 76% to 84% while multiple fistulas are present in 10.7% to 45% of the cases reported [1, 3–5]. In any case, a meticulous investigation is necessary as the detection of another fistula is a relatively frequent finding. In our case, only one communication between the LAD and PA was present and the fistula was easily ligated.

According to the American Cardiology Association guidelines for coronary artery fistulas, the intervention is absolutely indicated for large coronary artery fistulas regardless of the presence of symptoms while for small to moderate coronary artery fistulas it is performed after total evaluation of the patient's clinical status [6]. There are no definite criteria that determine the time of the intervention, although some researchers support certain methodologies. More specifically, Chang et al. have studied 29 patients with coronary artery-pulmonary artery communication with measurements from cardiac CT and found that right ventricle diameter/left ventricle diameter > 1.0 and CT score of right ventricular dysfunction more than 4.0 are two parameters that may determine the necessity for intervention [7]. Huang et al. have used fractional flow reserve to estimate the hemodynamic significance of the flow of the fistula by temporary occlusion of the fistula [8]. Such methods can be really beneficial in the management of coronary artery fistula. In our case, the surgery was inevitable and indicated in order to treat this life-threatening condition.

Throughout the literature, the clinical conditions associated with fistula LAD to PA vary. More specifically, the basic factors that affect the hemodynamic status of the patient are the steal phenomenon, which is usually combined with atheromatic coronary disease [9], and the shunt that affects the function of the involved structures, which may be the main cause for congestive heart failure in 20% of the patients [5]. There are cases that sudden death is reported due to LAD-PA fistula and ischemia is reported even in a patient without the presence of stenotic coronary artery as a consequence of extreme steal phenomenon [10, 11]. In this case report, PASP and increased right ventricle volume were found at the patient's admission while 2 years before these findings were absent. The cause of the development of pulmonary hypertension is probably related to the increase of the stenosis of the LAD leading to increased steal phenomenon towards the pulmonary artery. A very interesting point of this case is that the pulmonary hypertension progressed rapidly after a limit to coronary stenosis was reached. The almost total reverse of the pulmonary hypertension after the surgery and the following further improvement strengthen this hypothesis for the possible mechanism for the appearance and total reverse of pulmonary hypertension due to LAD-PA fistula combined with severe LAD stenosis.

## Data Availability

The data that support the findings of this study are available on request from the corresponding author. The data are not publicly available due to privacy or ethical restrictions.
